# Optimization of Bioactive Compound Extraction from Rose Myrtle Fruit (*Rhodomyrtus tomentosa*, (W.Ait), *Myrtaceae*) as the Antioxidant Source

**DOI:** 10.1155/2020/9105847

**Published:** 2020-04-14

**Authors:** Titik Ismandari, Sri Kumalaningsih, Susinggih Wijana, Siti Asma'ul Mustaniroh

**Affiliations:** ^1^Agrotechnology Program Study, Faculty of Agriculture, Borneo Tarakan University, Amal Lama Street No. 1, Tarakan, North Kalimantan 77115, Indonesia; ^2^Department of Agro-industrial Technology, Brawijaya University, Veteran Street, Ketawanggede, Lowokwaru, Malang, Indonesia

## Abstract

Rose myrtle fruit (*Rhodomyrtus tomentosa*, (W.Ait), *Myrtaceae*) is one of fruits widely found in Kalimantan. This fruit contains a bioactive compound that has a potential to be used as medicine. The aim of this study was to obtain optimal temperature and time of extraction in maintaining and protecting the bioactive compound in rose myrtle fruit extract by using water as solvent. This research applied the response surface method with central composite design for two factors, namely, *X*_1_ (temperature/°C) consisting of three levels: 70, 80, and 90°C and *X*_2_ (time/minute) which consisted of three levels of 60, 90, and 120 minutes. Research parameters included total phenol and antioxidant activity. Moreover, GC-MS was used for the characterization of the chemical compound component contained in rose myrtle fruit extract. Optimization of extraction condition resulted in an optimum temperature for extraction of 80.43°C and optimum time for extraction of 85 minutes with an optimum yield of total phenol of 73.77 mg/100 g fresh fruit and antioxidant activity of 1.0385 *µ*g/ml with desirability of 0.892 or 89.2%.

## 1. Introduction

Rose myrtle fruit (*Rhodomyrtus tomentosa*, (W.Ait), *Myrtaceae*) is one of the potential fruits in North Kalimantan, particularly in Tarakan City. The production of rose myrtle fruit ranged of 1–1.3 ton/ha [1]. Rose myrtle fruit contains a bioactive compound which provides benefit for health [2]. Moreover, there are also 19 phenolic compounds and gallic acid which has a potential to be an antioxidant source [3]. As mentioned by Lai et al. [4], 150 gram of dry rose myrtle fruit contains food fiber (69.94–87.43%), *α*-tocopherol (38.90–51.87% RDI), and linoleic acid (75.36% of total fatty acid).

Antioxidant is a bioactive compound with an ability to inhibit free radical and substrate oxidation. Free radical leads to chain reaction, resulting in cell damage, cellular aging, and chronic degenerative diseases such as cancer, diabetes, cardiovascular and neurovascular disease [5].

In general, fulfilment of antioxidant needed for the body is carried out using a synthetic antioxidant like 4-hexylresorcinol [6], yet several researchers revealed that the use of synthetic antioxidant succeeded in causing negative effect on health in addition to its toxicity [7]. Therefore, natural antioxidant becomes one of alternatives urgently required.

The component of bioactive compound in plants has an ability to reduce free radical [8]. Furthermore, as mentioned by Maskam et al. [9], rose myrtle fruit was able to inhibit DPPH of free radical by 62.13%, and this value was higher compared with antioxidant activity of mangosteen peel of 2.496 *μ*g/ml [10] and noni leaf (123.72 *µ*g/m) [11]. However, the use of rose myrtle plant until now, particularly its fruit in North Kalimantan, is very limited. This condition is due to the limitation of information on the used technique and benefit of the plant.

Until today, rose myrtle plant has not been maximally used and only considered as weed with quite high growth rate; thus, it exists as a pest plant that is difficult to control. The benefit of rose myrtle plant, especially its fruit, is not widely exposed, and this plant is commonly considered as weed in order not to harm cultivated plants.

Concerning the chemical composition, bioactive compound content, and antioxidant compound in rose myrtle fruit, it is necessary to conduct a study on the use of rose myrtle fruit as an antioxidant source by not causing damage of the bioactive compound through a simple method. The method applied was extraction using water as solvent. Water is an organic solvent that is safe, inexpensive, easy to obtain, and never been used in the extraction of rose myrtle fruit. The aim of this study was to obtain optimal temperature and time of extraction in maintaining and protecting the bioactive compound in rose myrtle fruit extract by using water as solvent.

## 2. Materials and Methods

### 2.1. Materials

Materials used in this study were rose myrtle fruits (*Rhodomyrtus tomentosa,* (W.Ait)*, Myrtaceae*.) obtained from Tarakan City, North Kalimantan, with criteria of physiologically ripe fruit and water as solvent. Tools used included a water bath, thermocouple, digital scale, and rotary evaporator.

### 2.2. Methods

The stages of this study are as follows: (1) sorting and washing, (2) reducing size, (3) extraction according to treatments, and (4) analysis of total phenolic content (GAE) and antioxidant activity of rose myrtle fruit extract. Sortation of rose myrtle fruit was performed by separating or sorting good fruits out of the damaged or defective fruit and other foreign object. Later, fruits were cleaned in running water, blended until smooth, and prepared to be extracted.

### 2.3. Extraction of the Bioactive Compound from Rose Myrtle Fruit

Extraction of the rose myrtle fruit bioactive compound was performed using the dekok method with water as solvent. This process was started with sortation activity, followed by rose myrtle fruit washing. Furthermore, clean fruits were weighed of 100 gram, thinly sliced at a size of ±1 mm, and blended. After that, extraction in water bath was carried out using water as solvent at a ratio of 5 : 1 (solvent: sample) at temperature and time according to treatments. The product of rose myrtle fruit extraction was filtered using a vacuum filter to obtain filtrate and dregs. Removal of solvent in filtrate was performed using a vacuum rotary evaporator at a temperature of 40°C for 1 hour in order to obtain the filtrate. The filtrate collected was further centrifuged at the speed of 5000 rpm for 10 minutes to precipitate dirt, resulting in a supernatant and pellet. The supernatant obtained was stored in a refrigerator to be analyzed further.

### 2.4. Experimental Design for Optimization of Bioactive Compound Extraction through the Response Surface Method (RSM)

The Response Surface Method was used to assess total yield and total phenol produced from extraction on two independent variables of extraction condition, namely temperature and time of extraction. The composition of the two independent variables was designed using central composite design. The point of temperature variable was 80°C, while the point obtained for variable of time was 90 minutes. The model of mathematical equation of the central composite model with 2 factors is

(1) $$\begin{array}{c} Y=\beta_{0}+\overset{2}{\underset{i=1}{\sum }}\beta_{i}X_{i}^{2}+\sum \overset{2}{\underset{i<j=1}{\sum }}\beta_{u}X_{i}X_{j}. \end{array}$$

In this term, Y is the response (yield), *β*0 is constant, *β*_i_, *β*_ii_, and *β*_ij_ are coefficients of independent variable (*X*), *X* is independent variable without code (for variable of time of extraction: temperature of extraction (*X*_1_) at level of 70, 80°C, and 90°C; time of extraction (*X*_2_) at level of 60, 90, and 120 minutes), and *ε* is random error. Level of independent variable (temperature and time) in this study is presented in Table 1 [12].

### 2.5. Analysis of Total Phenolic Content

Analysis of total phenol was performed through the spectrophotometry method by using the Folin–Ciocalteau reagent [13]. Total phenolic compound in rose myrtle fruit is expressed as Gallic Acid Equivalent (GAE). Gallic acid GAE is a common reference to measure the amount of phenolic compound in a material. Gallic acid is used as a standard. Calculation of this total phenol used the standard of concentration of 0; 1.5; 3; 6; and 8 ppm, with equation of standard curve *y* = 0.0956*x* + 0.0029 with *R*^2^ = 0.9989. Treatment of the sample was similarly made using the method of standard curve. Total phenol was determined based on the value of gallic acid equivalent.

### 2.6. Determination of Antioxidant Activity

Antioxidant activity of the rose myrtle fruit extract was determined in accordance with the method of DPPH which is based on the ability of sample in reducing DPPH stable-free radical, 1. 1, 1-diphenyl-2-picrylhydrazyl [14]. One ml of 0.5 mM DPPH was put into test tube and added with 50 *μ*l of rose myrtle fruit extract at various concentrations. Furthermore, 3.95 ml of ethanol was added. The sample was homogenized using vortex and left for 30 minutes. The concentration of rose myrtle fruit powder obtained was increased until it reached the value of IC_50_, which was the concentration that produced radical capture concentration of 50% compared to control through a linear regression line equation. Later, absorbance of the solution was read at a wavelength of 517 nm. Reading on the absorbance of control solution, which was without the addition of vitamin E, was also conducted.

### 2.7. Statistical Analysis

Statistical analysis was performed using software MINITAB Release 14. This analysis resulted in a coefficient influencing the model and graph from the observed response in the form of coefficient of regression, 3D response surface plot, and contour plot (using Design expert 7), to examine the model concerning the optimum extraction process [15].

## 3. Results and Discussion

The observation result of the value of total phenol and antioxidant activity (IC_50_) is presented in Table 2.

Table 2 presents the actual value and variable response of total phenol and antioxidant. Total phenol of observation result ranged from 36.629 mg/g to 81.158 mg/g, while the antioxidant activity (IC_50_) ranged between 1.02 *µ*g/ml and 2.0612 *µ*g/ml. Response of each variable is explained as follows.

### 3.1. Total Phenolic Content

**Figure 1:**
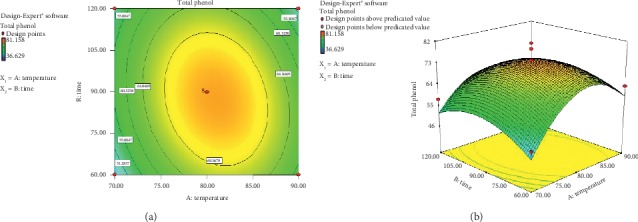
(a) Contour plot and (b) surface response which showed the effect of temperature and time of extraction on total phenol of rose myrtle fruit extract.

Determination of total antioxidant of foods from plants can be performed by measuring the concentration of total phenolic content using the Folin–ciocalteau reagent. The phenolic compound has an important role in preventing oxidation [16]. Moreover, John et al. [17] said that test of total phenol was aimed to determine the total phenolic compound contained in a sample; thus, it was expected that high concentration of phenolic compound content in the sample resulted in high antioxidant activity. According to the analysis result of model selection based on “*Sequential Model Sum of Squares,”* the selected model was quadratic for its value of 0.0031 (<5%) which showed that the chance error of model was less than 5%; thus, it significantly affected the response of total phenol. Furthermore, based on the measurement of *Lack of Fit*, the quadratic model was defined as “*suggested*”, which meant that the selected model obtained a value of 0.2981 or it was not significantly different since the *p* value > 5%; hence, it was concluded that the models were the appropriate models for the response of total phenol. The equation of RSM from the extraction process is as follows:

(2) $$\begin{array}{c} Y=-10200.172+244.7916\;X_{1}+23.3496\;X_{2}-0.1153583\;X_{1}X_{2}-1.448045\;X_{1}^{2}-0.0801856\;X_{2}^{2}, \end{array}$$

where *Y* is total phenol, *X*_1_ is temperature of extraction, and *X*_2_ is time of extraction. The equation shows that the response of total phenol will increase, directly proportional to the increasing temperature and time of extraction, reflected by the positive constant value. The value of *R*-square (*R*^2^) = 0.81752 indicated that the data were able to support the model for about 81.75%, which included the factor of temperature and time of extraction. The rest of 18.25% was affected by other factor excluded in the model. Other factors that influenced total phenol were the extraction method, solvent type, solvent composition, and plant species. The contour plot and visualization of response surface produced from the data of total phenol in the extraction process that used response surface test are presented in Figure 1(a) and 1(b).

As shown in Figure 1(a), the *x*-axis reflects the factor of temperature and the *y*-axis is for factor of time. The line within the contour plot is the value of total phenol response. Concerning the contour color, the red dot shows the highest value of total phenol response of 81.158 mg GAE/g, while the lowest value is indicated by red color in contour plot of 36.629 mg GAE/g. The shape of response surface produced from the interaction between these components is seen more clearly in the 3D graph as shown in Figure 1(b). In response surface, Figure 1(b), it is seen in the treatment value of extraction temperature and time that the total phenolic concentration of rose myrtle fruit extract increased along with the increasing temperature and time of extraction, and later, it was stable and tended to decrease afterward. According to Wazir et al. [18], the use of high temperature in the extraction process will increase solubility of cell wall or the bound phenolic compound due to cell element damage. Therefore, more phenolic compound is extracted. In addition, increase in temperature causes the pores of solids to expand, thus water as solvent will easily diffuse into the pores of solids of rose myrtle fruit extract and dissolve phenol. Hence, more phenol will be there to interact, and this leads to higher mass transfer of *solute,* namely from solid material to solvent.

Based on the analysis, it is known that the critical values for temperature and time were 80°C and 90 minutes, respectively. In that point, total phenol was predicted to reach 81.158 *µ*g/g. However, at a temperature above 80°C, phenolic concentration decreased due to the high temperature during the extraction process. Along with the increasing temperature, it is easier for phenol to exit the cell of rose myrtle fruit. Heating during extraction process also has a function to inactivate the enzyme of polyphenol oxidase [19]. The result mentioned above was in accordance with the study conducted by Susanti [20] that higher temperature applied in extraction process will lead to higher inactivation of polyphenol oxidase enzyme; thus, enzyme activity will be lower and phenol damage will be smaller. However, phenolic content will also be hampered due to the increasing temperature of extraction; thus, the amount of total phenol detected will reach the maximum peak and further will be constant and tended to decline. Moreover, as mentioned by Che Sulaiman et al. [21], phenolic compound will increase along with the increasing temperature and time of extraction, yet total phenol will decrease at high temperature since several phenolic compounds are sensitive to heat; hence, increasing temperature will decrease phenol. Furthermore, long extraction time may result in decomposition and phenolic oxidation [22, 23].

In addition to the effect of temperature and time of extraction, the use of solvent also determined the total phenol produced. In this study, extraction of rose myrtle fruits used water as solvent. Water is one of solvents that is safe and has quite high solubility on the extracted substrate. Water is a polar molecule and the extract of rose myrtle fruit is also polar; thus, extraction process produced phenol in high amount [24]. Total phenol in this study was found to be higher than the result of the previous study. In extraction of fresh rose myrtle fruit conducted by Lai et al. [25], solvent of *acetone*:water:*acetic acid* (50 : 49 : 1) resulted in a total phenol of 11.0 mg/100 g fresh fruit. Moreover, Zhao et al. [26] mentioned that total phenol in rose myrtle fruit dried using various drying methods and extracted using 80% *aceton*e as solvent was 15.57 mg/100 g DW.

### 3.2. Antioxidant Activity Content

Antioxidant is a bioactive compound that can inhibit free radical and substrate oxidation in which free radical may cause chain reaction that may result in cell damage, cellular aging, and chronic degenerative diseases, such as cancer, diabetes, cardiovascular, and neurovascular disease [5].

Result of Analysis of Variance of treatment on the antioxidant activity showed that the quadratic model selected had the value of F compute of 43.962 and *p* value = 0.0001, temperature obtained the value of *F* compute = 2.803 and *p*=0.138, and time had the value of *F* compute = 0.502 and *p*=0.501. The value of *p* < 0.05 indicated significant effect on response. Single factor of temperature and time of extraction resulted in the value of *p* of temperature = 0.0001 and the value of *p* of time = 0.0001 which meant that the value < 0.05; thus, both treatments significantly affected the response of antioxidant activity of rose myrtle fruit extract. The value of model deviation (*lack of fit*) obtained was 0.0001.

**Figure 2:**
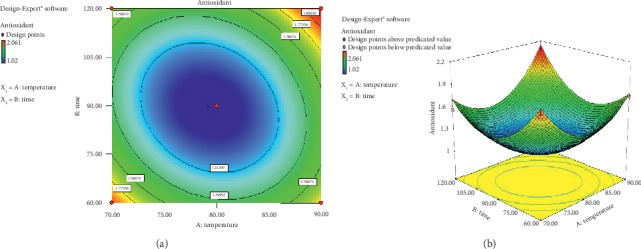
(a) Contour plot and (b) surface response which showed the effect of temperature and time of extraction on antioxidant activity of rose myrtle fruit extract.

The standard deviation obtained was 0.106 with *R*^2^ = 0.969. The value of R-square (coefficient of determination) in ANOVA of 0.969 reflected that the data were able to support the model for 96.90% which included the factor of temperature and time of extraction. The rest of 3.1% was influenced by other factors excluded in the model. Other factors affecting antioxidant activity were the extraction method, solvent type, solvent composition, and plant species. The value of *R* (*Adj R-Squared*) in ANOVA of 0.947 reflected that coefficient of correlation of 94.7% was obtained. Equation of RSM from the extraction process is as follows:

(3) $$\begin{array}{c} Y=36.52497-0.75309X_{1}-0.125881X_{2}+0.000509X_{1}X_{2}+0.004459X_{1}^{2}+0.0004779X_{2}^{2}, \end{array}$$

where *Y* is antioxidant activity, *X*_1_ is temperature of extraction, and *X*_2_ is time of extraction. The equation showed that antioxidant activity will increase, directly proportional to the increasing temperature and time of extraction as shown by the positive constant value. The value of *R*-square (*R*^2^) obtained = *R*^2^ = 0.969. The value of R-square (coefficient of determination) in ANOVA of 0.969 indicated that the data were able to support the model for 96.90% which included the factor of temperature and time of extraction. The rest of 3.1% was affected by other factors excluded in the model. Other factors affecting antioxidant activity were the extraction method, extraction type, solvent composition, and plant species. The contour plot and visualization of response surface from the data of antioxidant activity in the extraction process that applied the response surface method can be seen in Figure 2.

In Figure 2(a), red color of contour showed the highest value of antioxidant response of 2.0612 *µ*g/ml. The blue color indicated the lowest response value of 1.02 *µ*g/ml. The line consisted of dots in the graph of *contour plot* showed a combination of both components at different amounts which resulted in the same value of antioxidant response. The shape of response surface of the interaction between components can be seen more clearly in a three-dimension graph as shown in Figure 2(b). In term of curve trend, higher addition of temperature and time of extraction increased antioxidant value and further the value decreased after it passed the optimum treatment. This finding was in line with the study carried out by Zhao et al. [26] that increase in temperature and time of extraction will both increase and decrease antioxidant activity. It was due to the reason that heat will damage the cell tissue of plants extracted, thus increasing the amount of active component freed, yet the component will change and the amount will decrease along with the increasing temperature and time.

In the 3D graph, it is seen that antioxidant activity continued to increase until the temperature reached 80°C with an extraction time of 90 minutes. This increase was caused by the solubility of active compound on the material which was possible due to cell wall damage resulted from heating during the extraction process [27]. Moreover, according to Wang et al. [28], in addition to heat, high antioxidant activity was caused by water as solvent in the extraction process, in which antioxidant property in rose myrtle fruit is hydrophilic; thus, the extracted bioactive of antioxidant was quite high. A similar result was found by Tan et al. [23] that the use of polar solvent would generate polar extract which reflected active antioxidant activity.

Treatment of high temperature may result in the damage of cell wall and subcellular components of the plant cell to release large amount of active compound and produce strong free radical capturing component compared to fresh material. Hence, extraction at high temperature will cause tissue softening and the release of bound antioxidant component [29].

Based on the analysis result using DPPH reagent, the value of antioxidant activity of IC_50_ had a range between 1.02 *µ*g/ml and 2.0612 *µ*g/ml. This outcome showed that antioxidant value (IC_50_) of rose myrtle fruit extract for all treatment variations was included in the group of highly strong antioxidant of <50 Dg/ml, compared to the result of Cui et al. [30] who performed extraction using TFA and methanol as solvent and produced IC_50_ of 6.27 *µ*g/m and Maskam et al. [9] with extraction using solvents of water:methanol:chloroform:petrolium ether and produced IC_50_ of 107–250 *µ*g/ml, as well as Pingping et al.[31] with 95% ethanol as solvent that resulted in IC_50_ value of 10.97 *µ*g/ml. Therefore, the result of this study obtained better IC_50_.

### 3.3. Optimization of Total Phenolic Response and Antioxidant Activity

**Figure 3:**
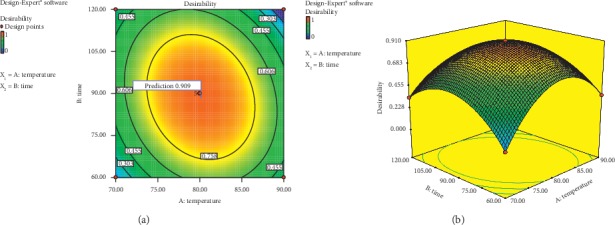
Graph of contour plot (a) and response surface (b) value of desirability.

The optimization result using *Design Expert Ver. 9 Trial* indicated that optimal condition recommended in the extraction process of rose myrtle fruit included an extraction temperature of 80.43°C and a time of 85.00 minutes. This treatment produced a response prediction in the form of total phenol of 73.77 mg/100 g fresh fruit and antioxidant activity of 1.0385 *µ*g/ml with *desirability* of 0.892 or 89.2%. The contour plot and response surface of prediction result are presented in Figure 3.

In Figure 3, it is seen that the value of *desirability* shown is within the lines inside the contour plot. *Desirability* indicates the desired scale for each response and determines the degree of desirability for the optimal solution result. The range of scale for *desirability* value was between 0 and 1, in which 0 reflected that the response was totally undesirable, while 1 showed that the response was completely wanted [15]. This study obtained a *desirability* value of 0.892 or 89.2%, in which if the number was closer to one, the desirability value for optimization will also increase.

Result of the study on the parameter of total phenol and antioxidant activity has a similar trend of graph. At higher temperature and longer time of extraction, the value of both parameters will increase, yet the value will decline along with the increasing temperature and time of extraction. Effect of heating does not only increases total phenol and antioxidant activity but also decreases it. Declining total phenol and antioxidant activity occurred at temperature above 80°C and a time longer than 90 minutes. Chew et al. [32] mentioned that longer time extraction and higher temperature will result in oxidation of the phenolic compound and antioxidant activity.

Based on the result of research, the Dekok extraction method that used water as solvent has a prospect to be further developed at a larger scale since it has a simple, safe, and easy-to-apply technique (*user friendly*) [33]. Several researches performed rose myrtle extraction using supercritical carbon dioxide [34], *reflux* [4], and ultrasonic [25]. However, besides some advantages gained from the technique, particular and expensive equipment are required before extraction was performed; therefore, it is not appropriate to be applied in small- and medium-scale industry (SMEs), Vongsak et al. [33], particularly in North Kalimantan.

## 4. Conclusion

This study showed that Surface Response is an effective method to optimize the condition of rose myrtle fruit extraction. The model of response surface was verified statistically through ANOVA. The calculation result of all independent variables had significant effects (*p* < 0.05) on all responses. The value of *R*^2^ of total phenol = 0.81752 and *R*^2^ of antioxidant activity = 0.969, and this result showed that the quadratic polynomial model applied was accurate in analyzing interaction of all parameters in study. The optimum condition in protecting bioactive compound in rose myrtle fruit was 80.43°C of temperature and time extraction of 85.00 minutes. This treatment produced total phenol of 73.77 mg/100 g fresh fruit and the antioxidant activity of 1.0385 µg/ml with desirability of 0.892 or 89.2%, in which the desirability of optimization will be higher if the desirability value is closer to one. Furthermore, to investigate the effect of the extraction process on the content of bioactive compound in rose myrtle fruit extract, it is necessary to conduct further research on the content of bioactive compound in rose myrtle fruit after the extraction process.

## Figures and Tables

**Table 1:** Level of independent variable, code, and optimized value.

Independent variable	Unit	−1	+1	−*α*	+*α*
Temperature (*X*_1_)	°Celcius	70	90	65.9	94.14
Time (*X*_2_)	Minute	60	120	47.57	132.4

**Table 2:** Matrix of factor and level in optimization of rose myrtle fruit extraction using central composite design.

Coded factor value		Actual factor value		Total phenolic response (mg GAE/g)	Antioxidant response (IC_50_) *µ*g/ml
Temperature of extraction (°C)	Time of extraction (minute)	(*X*_1_)	(*X*_2_)		
0	0	80	90	74.151	1.02
−1	−1	70	60	49.845	1.872
−1	+1	70	120	57.726	1.57
0	0	80	90	81.158	1.034
−1.41	0	65.9	90	36.629	1.964
0	−1.41	80	47.57	57.538	1.908
+1	+1	90	120	57.377	2.059
0	+1.41	80	132.4	50.879	2.054
0	0	80	90	78.764	1.031
0	0	80	90	69.087	1.028
0	0	80	90	65.367	1.03
+1	−1	90	60	63.339	1.75
+1.41	+1	94.14	90	42.733	2.0612

## Data Availability

The data used in this study are included within the article in Table 2.
